# Supplementary material for: Masked graph modeling for molecule generation
Source: Nat Commun. 2021 May 26;12:3156. doi: 10.1038/s41467-021-23415-2 (PMC8155025; doi:10.1038/s41467-021-23415-2)
Supplement: Supplementary file 1 — Supplementary Information [file 41467_2021_23415_MOESM1_ESM.pdf]

Supplementary Information: Masked Graph Modeling for Molecule Generation

Mahmood et al

A Supplementary Discussion

Related Work

In-Silico Molecular Generation Many of the previously proposed generative models of molecules focused on extending the variational autoencoder (VAE) for molecular generation. Gómez-Bombarelli et al. [1] proposed the first variational autoencoder (VAE) [2] based model for generating molecules in their SMILES representations. To address the issue of VAEs generating syntactically invalid SMILES strings, Kusner et al. [3] explicitly added the grammar of SMILES strings to VAEs for molecule generation. Wang et al. [4], Guimaraes et al. [5] and Cao and Kipf [6] used a generative adversarial network (GAN) [7] to build a generative model of small molecular graphs. Unlike most recent work that has focused on neural network-based approaches, Jensen [8] showed that genetic algorithms based on Monte Carlo Tree Search (MCTS) could be competitive on the task of molecular generation.

Masked Language Models Masked language models, such as BERT [9], have been shown to bring significant improvements to a variety of discriminative language understanding tasks such as question answering [10, 11] and natural language inference [12, 13]. Wang and Cho [14], Ghazvininejad et al. [15] and Mansimov et al. [16] proposed ways to generate text directly from trained masked language models. Wang and Cho [14] proposed the use of Gibbs sampling, and Mansimov et al. [16] proposed the use of adaptive Gibbs sampling approaches for effective text generation using masked language models. Ghazvininejad et al. [15] used conditional masked language models for parallel decoding in machine translation. They first predict all target words in parallel, and then repeatedly mask out and regenerate the subset of words that the model is least confident about for a fixed number of iterations. In parallel to the work investigating masked language models for text generation, Welleck et al. [17], Stern et al. [18] and Gu et al. [19] proposed methods for non-monotonic sequential text generation. Although these methods could be applied for generating molecular graphs in flexible ordering, there has not been work empirically validating this. Due to the popularity of masked language models in natural language processing tasks, there has been recent work investigating a similar approach for learning graph representations. Hu et al. [20] investigated the transfer to downstream tasks of graph neural networks that were trained to predict the masked node and edge attributes of graphs. Maziarka et al. [21] proposed the molecule attention transformer architecture that was pretrained to predict masked input nodes and investigated its transfer to downstream property prediction tasks. Unlike our work, neither Hu et al. [20] nor Maziarka et al. [21] investigated ways of generating novel molecular graphs with their trained models.

Effect of Generation Hyperparameters on Generation Quality

We analyze the effect of changing the masking rate and graph initialization on generation quality. In order to do so, we must choose results corresponding to a certain number of generation steps for each combination of masking rate and initialization. We therefore evaluate samples at intermediate steps of the generation process, as shown in Supplementary Figure 1, to determine how the values of the evaluation metrics change as the number of generation steps increases.

For training initialization (Supplementary Figures 1a and 1c), the initialized molecules have perfect validity, uniqueness, KL and Fréchet scores, and zero novelty score. As generation proceeds, changes are made to the training molecules, yielding some invalid molecules, so the validity decreases. Some of the changes yield new, valid molecules, so the novelty increases. These molecules are less similar to the dataset distributions than the training molecules are themselves, so the KL and Fréchet scores decrease. On the other hand, for marginal initializations (Supplementary Figures 1b and 1d), the initialized molecules are less likely to be valid or similar to the dataset molecules. The probability of obtaining duplicate molecules is low as well. Over time, the molecules converge to valid structures similar to the dataset molecules, so the validity, KL and Fréchet scores increase. For both training and marginal initializations, different initialized molecules may converge to the same molecule over time, lowering uniqueness.

For all configurations and all metrics, the slope of the score with respect to the number of generation steps tends to flatten over time. When presenting the results of our model for different masking rates and initializations, we use the benchmark scores at the final generation step.

We now use these results to analyze the effect of changing the masking rate and graph initialization for generation in Supplementary Table 1. On QM9, we find that using marginal initialization leads to slightly higher validity and novelty scores however with lower KL-divergence and Fréchet ChemNet Distance scores compared with using training initialization. When using marginal initialization, the masked graph model generates marginally more novel molecules at the expense of not capturing the properties of dataset molecules as well. On ChEMBL, the marginal initialization strategy results in validity scores close to 0, which is why we only consider the training initialization strategy in Supplementary Table 1. On both QM9 and ChEMBL, novelty increases significantly when increasing the masking rate while the validity, KL-divergence and Fréchet Distance scores drop.

Close observation of the results in Supplementary Table 1 suggests that the choice of masking rate and initialization strategy impacts the balance among the five metrics. Most significantly, increasing the masking rate results in a higher novelty score, and lower KL-divergence and Fréchet Distance scores. We can trade off between different metrics as desired by adjusting the initialization and masking rate.

Selecting Best Unconditional Generation Results

We have shown that the GuacaMol benchmark metrics are correlated and that our model can efficiently trade these metrics off against each other. Thus we cannot say that one generation strategy definitively outperforms another unless it achieves a higher score on each of the five metrics. However, for the sake of comparison with baseline models, we pick one generation strategy as follows: we select results from Supplementary Table 1 for each dataset corresponding to the highest geometric mean among all five metrics.

For QM9, the ‘best’ MGM results correspond to training initialization with a 10% masking rate. For ChEMBL, the ‘best’ MGM results correspond to training initialization with a 1% masking rate.

Effect of Validation Loss on Generation Quality

To determine whether validation loss is a suitable proxy for generation quality, we carry out generation from different training checkpoints of our ‘best’ QM9 model. During training, we carried out a hyperparameter search to find the configurations with the lowest validation loss, which we used as the criterion to select the best model for generation. The experiments in this subsection explore whether this choice is justified.

Supplementary Figure 2 shows the values of all five benchmark metrics corresponding to different loss values (i.e., different checkpoints) of our model. In general, as the validation loss increases, the metrics’ values decrease. We attribute the decrease in validity to the fact that a less well-trained model is less likely to have learned enough about the relationship between different parts of a graph to predict masked components that respect the chemical constraints inherent in this type of data. The increase in novelty and decrease in KL and Fréchet scores are explained by better-trained models being more likely to predict masked components from the most similar context in the training/validation data. Occasionally this causes our model to generate an exact copy of a molecule from the training dataset, lowering the novelty; in general, it produces molecules whose local neighborhoods are similar to those of molecules in the training/validation data, thereby increasing the KL and Fréchet scores. The sharp decrease in novelty and uniqueness as the loss increases from 1.17 to 1.65 can be attributed to the low validity, as GuacaMol implicitly penalizes all metrics when the validity drops below 0.5.

We conclude that selecting the model with the lowest validation loss for generation is a reasonable strategy. This implies that using more powerful graph neural networks within our *masked graph modeling* framework could improve generation quality. Finding model architectures that lower the validation loss is a good direction for future work.

B Supplementary Tables

Dataset	Mask Rate	Graph Init	Valid	Uniq	Novel	KL Div	Fréchet Dist
QM9	10%	train	0.886	0.978	0.518	0.966	0.842
	10%	marginal	0.922	0.972	0.568	0.930	0.645
	20%	train	0.678	0.988	0.789	0.901	0.544
	20%	marginal	0.719	0.982	0.792	0.893	0.529
ChEMBL	1%	train	0.849	1.000	0.722	0.987	0.845
	5%	train	0.558	1.000	0.952	0.869	0.396

Supplementary Table 1: **Effect of varying masking rate and graph initialization on the benchmark results for our masked graph model on QM9 and ChEMBL.**

d_0	MPNNs	Layers per MPNN	Batch Size	Learning Rate	LR Decay	Validation Loss
2048	1	4	100	0.0005	no	0.29
2048	1	4	800	0.0005	no	0.20
**2048**	**1**	**6**	**512**	**0.0001**	**no**	**0.12**
2048	1	6	512	0.0005	no	0.17
2048	1	6	512	0.005	yes	0.38
2048	1	6	1024	0.0005	no	0.16
2048	1	8	50	0.0005	no	0.32
2048	1	8	100	0.0005	no	0.23
2048	1	8	400	0.0005	no	0.19
2048	1	16	25	0.0005	no	0.40
2048	1	16	100	0.0005	no	0.23
2048	2	2	400	0.0005	no	0.22
2048	2	3	512	0.005	yes	0.38
2048	2	4	400	0.0005	no	0.17
4096	1	4	400	0.0005	no	0.28
4096	1	6	512	0.005	yes	1.00
4096	1	6	1024	0.0001	no	0.15
4096	1	6	1024	0.0005	no	0.19
4096	1	6	2048	0.0005	no	0.19

Supplementary Table 2: **Hyperparameter configurations and corresponding validation set loss on the ChEMBL dataset.** The rows are arranged in ascending order, greedily by column from left to right. LR decay stands for learning rate decay and corresponds to decreasing the learning rate to a minimum of 0.0005 by halving the current learning rate every 204,800 data points. The hyperparameter configuration corresponding to the lowest loss is given in bold font and was used to generate the ChEMBL results presented in the paper.

C Supplementary Figures

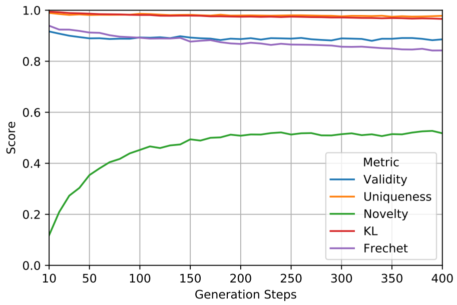

(a) Training initialization, 10% masking rate

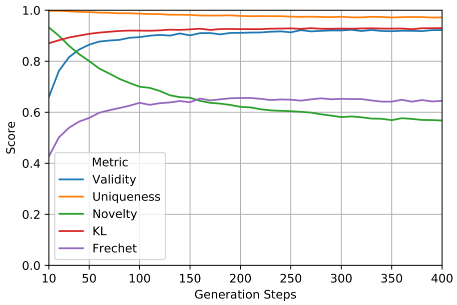

(b) Marginal initialization, 10% masking rate

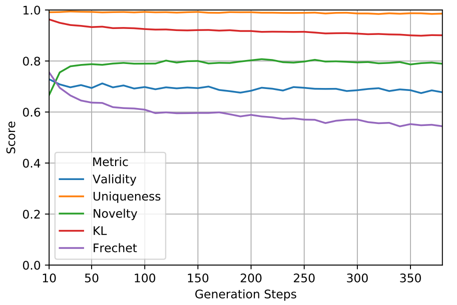

(c) Training initialization, 20% masking rate

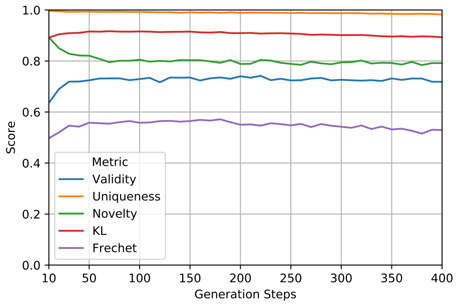

(d) Marginal initialization, 20% masking rate

Supplementary Figure 1: **Plots of generation scores as a function of number of generation steps for each initialization and masking rate on QM9.**

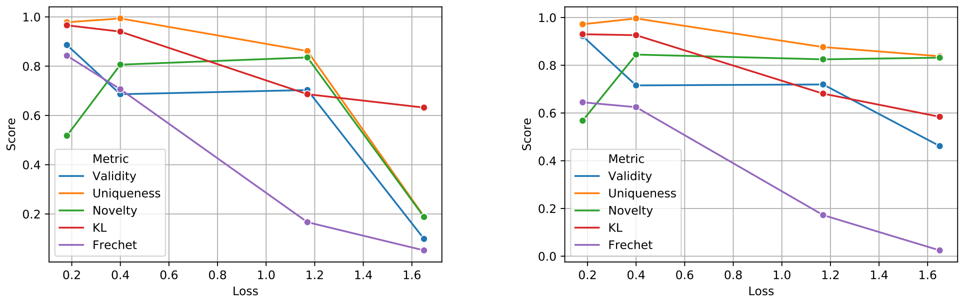

(a) Training Initialization (b) Marginal Initialization

Supplementary Figure 2: **Benchmark metric results on QM9 corresponding to our model's checkpoints corresponding to different validation loss values.** A masking rate of 10% was used.

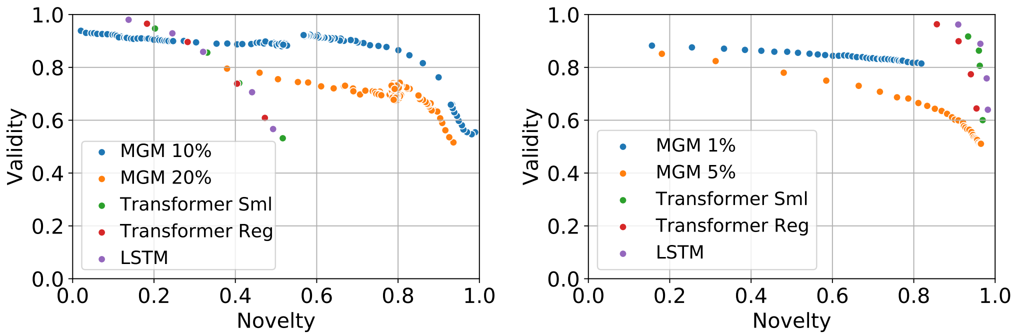

(a) QM9 (b) ChEMBL

Supplementary Figure 3: **Plots of validity against novelty, two anti-correlated metrics from the GuacaMol [22] distribution-learning benchmark.** The plots are generated in the same way as for Figure 1 in the main text.

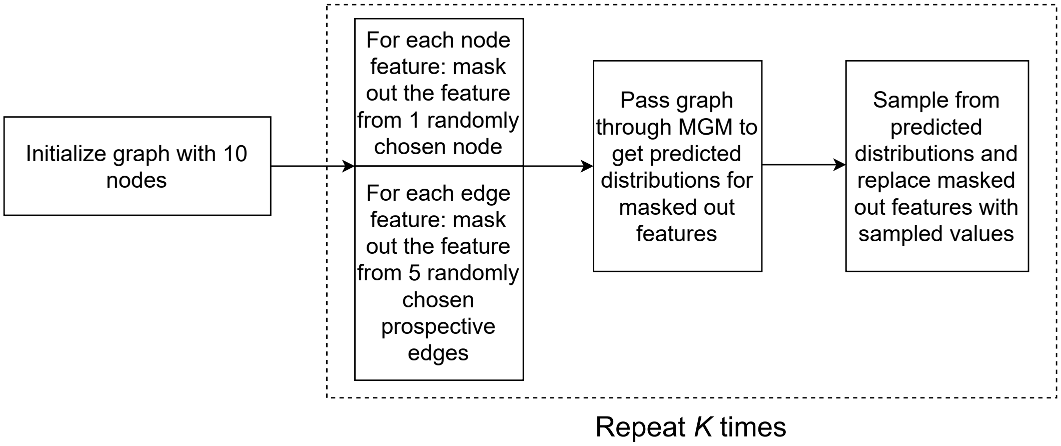

Supplementary Figure 4: **Schematic for unconditional generation with an initial graph with 10 nodes and a 10% masking rate.** The initial graph can either be taken from the training set (training initialization) or initialized using the training set distribution (marginal initialization). At each of the K sampling iterations, $\frac{10}{100} * 10 = 1$ node and $\frac{10}{100} * \frac{10(10-1)}{2} \approx 5$ prospective edges are masked out and replaced.

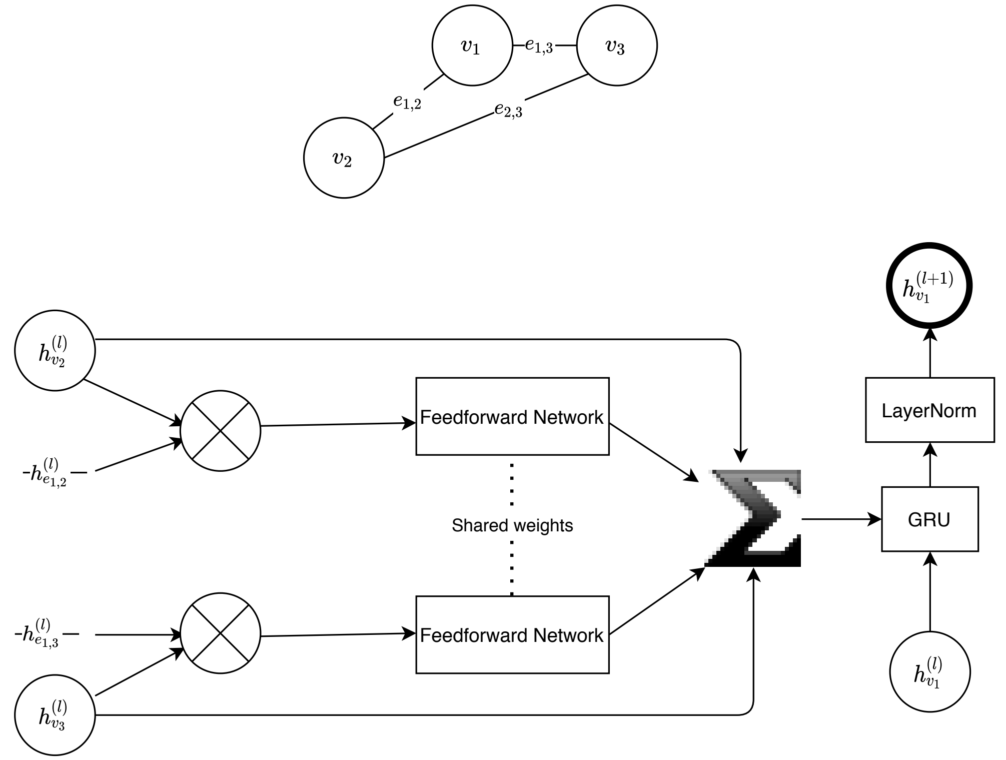

(a) Node Update Step. The diagram shows the calculation of the updated representation of node v_1 in the graph at the top of the figure. $\otimes$ denotes elementwise multiplication.

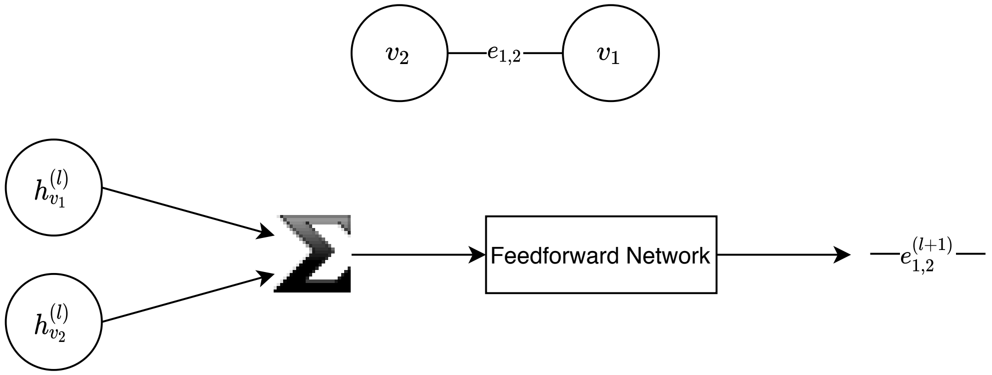

(b) Edge Update Step. The diagram shows the calculation of the updated representation of edge $e_{1,2}^{(l+1)}$ in the graph at the top of the figure.

Supplementary Figure 5: **MPNN update steps**

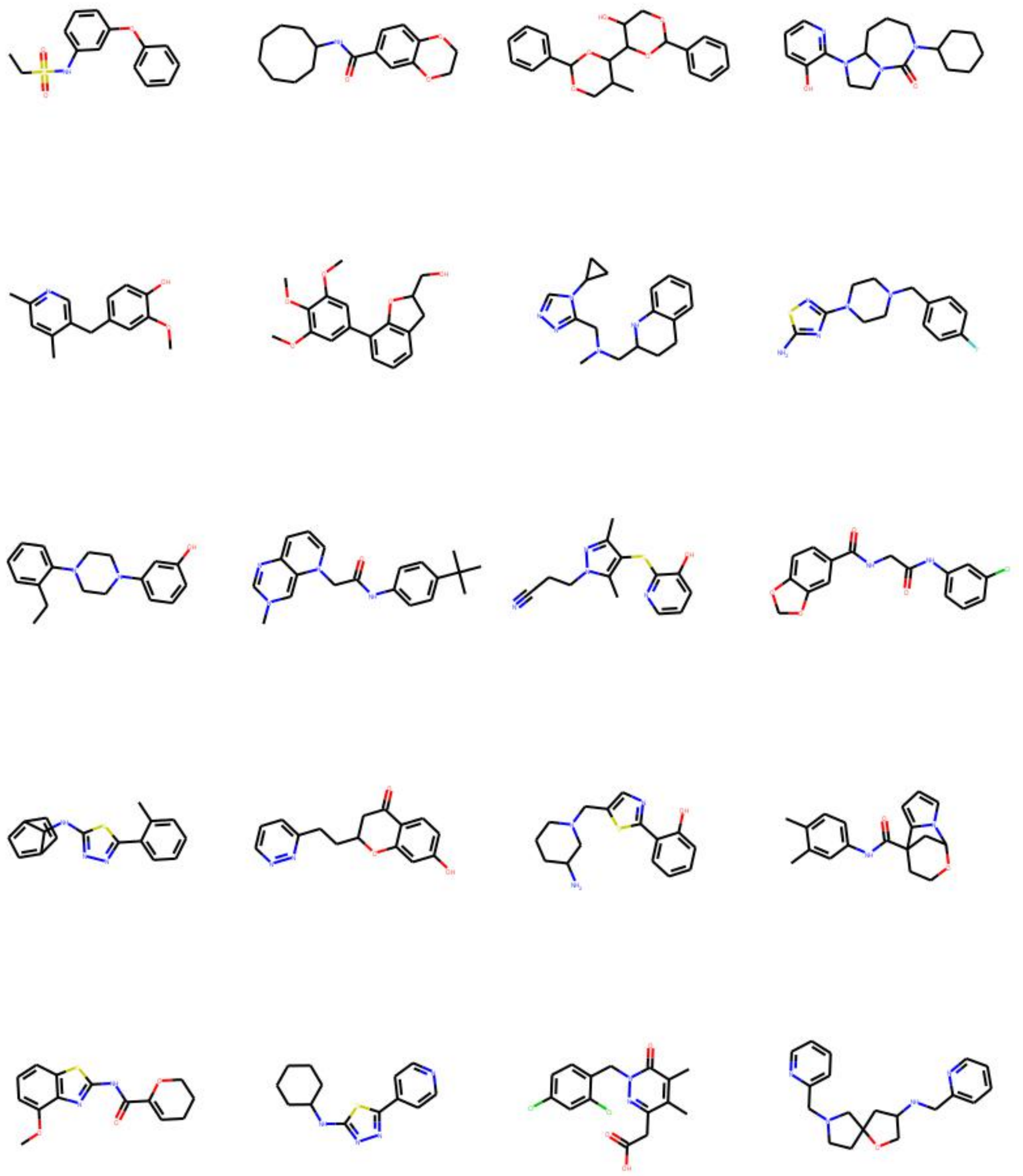

Supplementary Figure 6: **A selection of unconditionally generated novel molecules from ChEMBL.** The molecules are randomly chosen from the subset of novel generated molecules with QED score > 0.9.

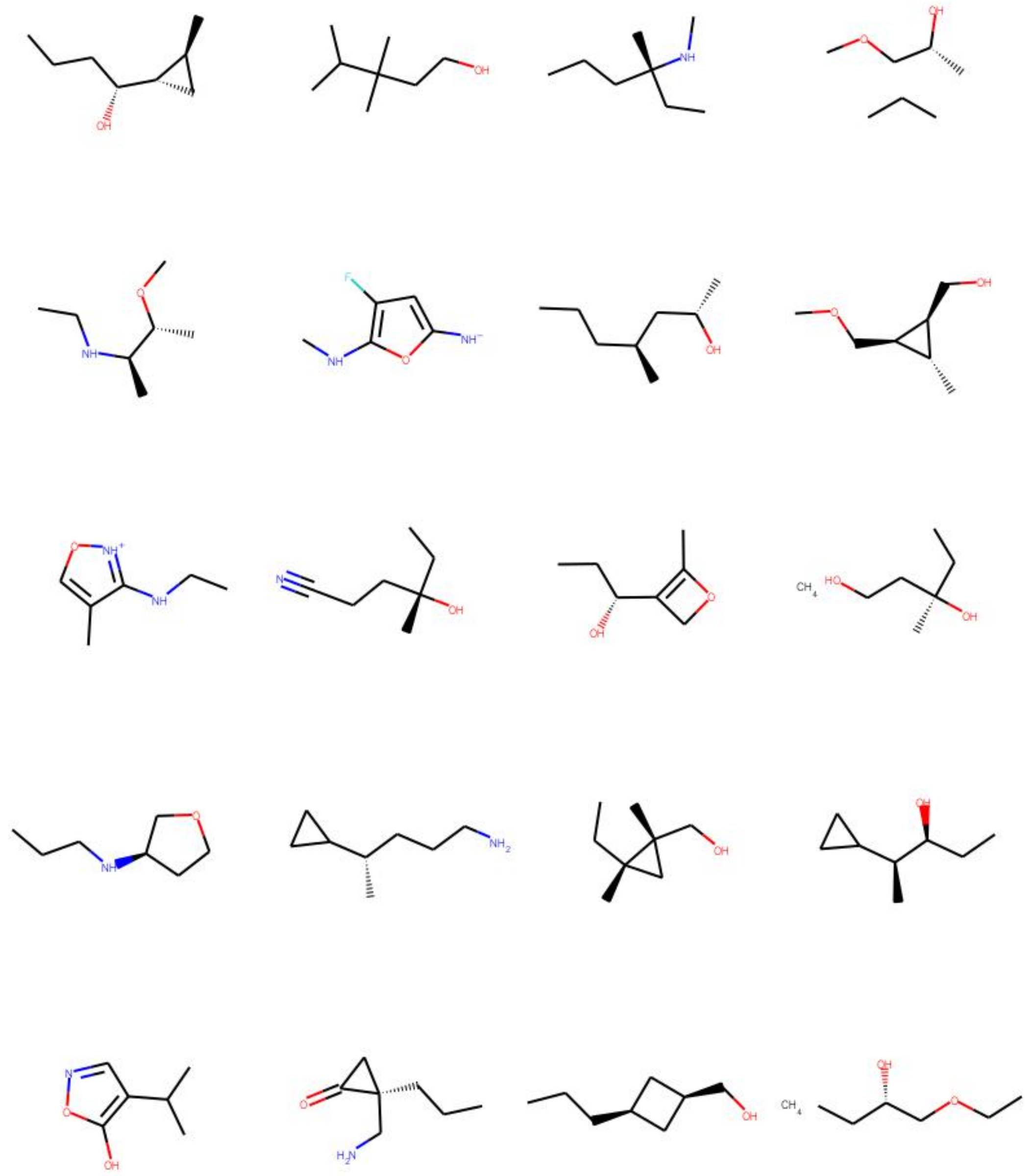

Supplementary Figure 7: **A selection of unconditionally generated novel molecules from QM9.** The molecules are randomly chosen from the subset of novel generated molecules with QED score > 0.6.

Supplementary References

[1] Rafael Gómez-Bombarelli, David Duvenaud, José Miguel Hernández-Lobato, Jorge Aguilera-Iparraguirre, Timothy D. Hirzel, Ryan P. Adams, and Alán Aspuru-Guzik. Automatic chemical design using a data-driven continuous representation of molecules. *arXiv preprint 1610.02415*, 2016.

[2] Diederik P. Kingma and Max Welling. Auto-encoding variational bayes. *arXiv preprint 1312.6114*, 2013.

[3] Matt J. Kusner, Brooks Paige, and José Miguel Hernández-Lobato. Grammar variational autoencoder. In *ICML*, 2017.

[4] Hongwei Wang, Jia Wang, Jialin Wang, Miao Zhao, Weinan Zhang, Fuzheng Zhang, Xing Xie, and Minyi Guo. Graphgan: Graph representation learning with generative adversarial nets. *CoRR*, abs/1711.08267, 2017. URL `http://arxiv.org/abs/1711.08267`.

[5] Gabriel Lima Guimaraes, Benjamin Sanchez-Lengeling, Pedro Luis Cunha Farias, and Alán Aspuru-Guzik. Objective-reinforced generative adversarial networks (ORGAN) for sequence generation models. *CoRR*, abs/1705.10843, 2017. URL `http://arxiv.org/abs/1705.10843`.

[6] Nicola De Cao and Thomas Kipf. Molgan: An implicit generative model for small molecular graphs. *arXiv preprint 1805.11973*, 2018.

[7] Ian J. Goodfellow, Jean Pouget-Abadie, Mehdi Mirza, Bing Xu, David Warde-Farley, Sherjil Ozair, Aaron C. Courville, and Yoshua Bengio. Generative adversarial nets. In *NIPS*, 2014.

[8] Jan H. Jensen. Graph-based genetic algorithm and generative model/monte carlo tree search for the exploration of chemical space. 2018.

[9] Jacob Devlin, Ming-Wei Chang, and Kristina Toutanova Kenton Lee. Bert: Pre-training of deep bidirectional transformers for language understanding. In *NAACL*, 2019.

[10] Pranav Rajpurkar, Jian Zhang, Konstantin Lopyrev, and Percy Liang. Squad: 100, 000+ questions for machine comprehension of text. *ArXiv*, abs/1606.05250, 2016.

[11] Pranav Rajpurkar, Robin Jia, and Percy Liang. Know what you don't know: Unanswerable questions for squad. *ArXiv*, abs/1806.03822, 2018.

[12] Samuel R. Bowman, Gabor Angeli, Christopher Potts, and Christopher D. Manning. A large annotated corpus for learning natural language inference. *ArXiv*, abs/1508.05326, 2015.

[13] Adina Williams, Nikita Nangia, and Samuel R. Bowman. A broad-coverage challenge corpus for sentence understanding through inference. *ArXiv*, abs/1704.05426, 2018.

[14] Alex Wang and Kyunghyun Cho. Bert has a mouth, and it must speak: Bert as a markov random field language model. *arXiv preprint arXiv:1902.04094*, 2019.

[15] Marjan Ghazvininejad, Omer Levy, Yinhan Liu, and Luke Zettlemoyer. Mask-predict: Parallel decoding of conditional masked language models. *arXiv preprint arXiv:1904.09324*, 2019.

[16] Elman Mansimov, Alex Wang, and Kyunghyun Cho. A generalized framework of sequence generation with application to undirected sequence models. *arXiv preprint arXiv:1905.12790*, 2019.

[17] Sean Welleck, Kianté Brantley, Hal Daumé, and Kyunghyun Cho. Non-monotonic sequential text generation. In *ICML*, 2019.

[18] Mitchell Stern, William Chan, J. Kiros, and Jakob Uszkoreit. Insertion transformer: Flexible sequence generation via insertion operations. In *ICML*, 2019.

[19] Jiatao Gu, Qi Liu, and Kyunghyun Cho. Insertion-based decoding with automatically inferred generation order. *Transactions of the Association for Computational Linguistics*, 2019.

[20] Weihua Hu, Bowen Liu, Joseph Gomes, Marinka Zitnik, Percy Liang, Vijay S. Pande, and Jure Leskovec. Strategies for pre-training graph neural networks. *arXiv preprint arXiv:v*, 2019.

[21] Lukasz Maziarka, Tomasz Danel, S. Mucha, K. Rataj, J. Tabor, and Stanislaw Jastrzebski. Molecule attention transformer. *ArXiv*, abs/2002.08264, 2020.

[22] Nathan Brown, Marco Fiscato, Marwin H.S. Segler, and Alain C. Vaucher. Guacamol: Benchmarking models for de novo molecular design. *arXiv preprint 1811.09621*, 2018.
